# Peroxiredoxin 4 protects against ovarian ageing by ameliorating d-galactose-induced oxidative damage in mice

**DOI:** 10.1038/s41419-020-03253-8

**Published:** 2020-12-11

**Authors:** Xiuru Liang, Zhengjie Yan, Weiwei Ma, Yi Qian, Xiaofei Zou, Yugui Cui, Jiayin Liu, Yan Meng

**Affiliations:** The State Key Laboratory of Reproductive Medicine, The Center for Clinical Reproductive Medicine, the First Affiliated Hospital of Nanjing Medical University, Nanjing, Jiangsu Province China

**Keywords:** Infertility, Experimental models of disease

## Abstract

Peroxiredoxin 4 (Prdx4), a member of the Prdx family, is a vital ER-resident antioxidant in cells. As revealed in our previous study, Prdx4 expression was detected in ovarian granulosa cells and was closely related to ovarian function. This research aimed to explore the effect and underlying molecular mechanism of the protective role of Prdx4 against d-gal-induced ovarian ageing in mice. The d-gal-induced ovarian ageing model has been extensively used to study the mechanisms of premature ovarian failure (POF). In this study, adult Prdx4^−/−^ and wild-type mice were intraperitoneally injected with d-gal (150 mg/kg/day) daily for 6 weeks. Ovarian function, granulosa cell apoptosis, oxidative damage and ER stress in the ovaries were evaluated in the two groups. Ovarian weight was significantly lower, the HPO axis was more strongly disrupted, and the numbers of atretic follicles and apoptotic granulosa cells were obviously higher in Prdx4^−/−^ mice. In addition, Prdx4^−/−^ mice showed increased expression of oxidative damage-related factors and the ovarian senescence-related protein P16. Moreover, the levels of the proapoptotic factors CHOP and activated caspase-12 protein, which are involved in the ER stress pathway, and the level of the apoptosis-related BAX protein were elevated in the ovaries of Prdx4^−/−^ mice. Thus, d-gal-induced ovarian ageing is accelerated in Prdx4^−/−^ mice due to granulosa cell apoptosis via oxidative damage and ER stress-related pathways, suggesting that Prdx4 is a protective agent against POF.

## Introduction

Infertility is a prevalent disorder worldwide and affects 15% of reproductive-aged couples^1^. Ovarian ageing is a major factor affecting female fertility due to depletion of ovarian follicles and loss of ovarian cyclicity^2^. An accumulated body of data implies that ovarian oxidative damage can result in severe ovarian dysfunctions^3^. d-galactose (d-gal) treatment in mice results in excessive reactive oxygen species (ROS) production and advanced glycation end product (AGE) accumulation in the brain, myocardium, liver, kidneys, muscles, vessels and ovaries^4–10^. Accumulation of ROS and AGE are causes of ageing that decrease ovarian function^11^. Because the accelerated ageing processes in the d-gal-induced ageing model are highly similar to those occurring during the course of ageing in humans, this model is widely used to explore the mechanisms of premature ovarian failure (POF).

Previous studies have suggested that an increased production of toxic metabolites caused by ageing-related ROS accumulation principally induces the apoptosis of ovarian granulosa cells^12^. Substantial amounts of data indicate that many antioxidants and antioxidant enzymes that defend cells against ROS may promote follicular development and survival^13^. The relationship of endoplasmic reticulum (ER) stress with oxidative stress is a vicious cycle and co-mediates apoptotic processes in cells^14^. ER stress is involved in granulosa cell apoptosis during follicular atresia^15^. Oxidative damage and ER stress are two important pathways of apoptosis that are involved in the occurrence and development of many ageing-related diseases.

Peroxiredoxin 4 (Prdx4) is an important ER-localised peroxiredoxin that eliminates H_2_O_2_ and promotes oxidative protein folding^16^. Prdx4 helps prevent steatosis progression, metabolic syndrome, inflammatory reactions and apoptotic activity by suppressing local and systemic oxidative stress. In reproduction, Prdx4 knockout leads to increased spermatogenic cell death via oxidative stress in mice^17^. Our published data show that Prdx4 is expressed in ovarian granulosa cells^18^. Moreover, we have demonstrated that Prdx4 expression is higher in middle-aged mice than either pubescent or aged groups of mice, and that Prdx4 can be detected at lower levels in ovaries from premenopausal women compared with young women^19^. These observations indicate that Prdx4 may have a protective effect on ovarian function. Additionally, the latest research with single-cell transcriptome sequencing technology analysis of ovaries from young and old cynomolgus monkeys identified Prdx4 as a new molecular marker of granulosa cell ageing, which is consistent with our results^20^.

However, it remains unclear if and how this factor may be involved in ovarian ageing. Considering the critical role of Prdx4 in counteracting ROS and ER stress, we postulated that Prdx4 plays a protective role against oxidative damage and ER stress in a mouse ageing model. In this study, we used d-gal to accelerate ovarian ageing to determine the effect of Prdx4 on ovarian function. Mature wild-type (WT) and Prdx4 knockout (Prdx4^−/−^) mice were injected with d-gal, and ovarian function, oxidative damage-related factors, and ER stress were compared.

## Materials and methods

### Animals and treatment

Female C57BL/6 mice were obtained from the Nanjing Biomedical Research Institute of Nanjing University, Nanjing, China. Knockout mice were generated by the CRISPR-associated Cas9 nuclease (CRISPR/Cas9) genome editing technique^21^. We designed and produced two sgRNAs targeting Prdx4 exons 1–7 (E1-E7) with the CRISPR Design Tool (http://crispr.mit.edu/) developed by Massachusetts Institute of Technology (sgRNA1 forward: 5′-AGCCAGCTAAAACGGCGCGCTGG-3′, reverse: 5′-CCTTGATCAGTAAATATCTTTGG-3′; sgRNA2 forward: 5′-GGGGCGGAGCAAGTCTCGCGAGG-3′, reverse: 5′-CACAATGACCTTTATTGAAGG-3′). To obtain Prdx4-positive heterozygous transgenic mice (F1 generation), F0 mice were mated with C57BL/6 mice, and neonates were examined by PCR and sequencing. Prdx4^−/−^ mice were viable and could mature to give birth.

This study aimed to investigate the effect of d-gal-induced acute oxidative stress on ovarian function in Prdx4^−/−^ and WT mice at sexual maturity. Acute oxidative stress is a widely recognised cause of ageing that leads to gradual damage to ovarian function, and d-gal-induced oxidative damage is widely used to establish mouse models of ageing to explore the mechanisms underlying ovarian ageing. In our preliminary study, no differences in physiological outcomes or ovarian function were observed between Prdx4^−/−^ mice and WT mice after intraperitoneal injection of saline. Considering the limited number of Prdx4^−/−^ mice, we used WT mice to determine the appropriate concentration to establish the acute oxidative stress mouse model. All 5-month-old mice were allowed to acclimatise for 1 week. Previous studies have reported the generation of POF model mice by daily intraperitoneal injections of d-gal (200 mg/kg/day)^22^. To avoid excessive stress, twenty WT C57BL/6 female mice, aged 5 months, were randomly divided into the following 4 independent groups (5 mice per group): saline, low-dose (d-gal, 50 mg/kg/day), medium-dose (d-gal, 100 mg/kg/day) and high-dose (d-gal, 150 mg/kg/day). All mice were injected daily for 42 days to determine the appropriate d-gal concentration for ovarian ageing induction.

The mice in this study were then divided into two groups of five mice per group: the WT and Prdx4^−/−^ groups. Mice in these two groups were intraperitoneally injected daily with the appropriate d-gal concentration for 42 days. All experiments involving animals were approved by the Institutional Animal Care and Use Committee (IACUC) of Nanjing Medical University, and the procedures were conducted in accordance with the approved guidelines.

### Oestrous cycles

Oestrous cycles were monitored by vaginal smear at 6 weeks after d-gal treatment for 12 consecutive days to evaluate the oestrous cycle stages of the mice (*n* = 5 per group)^23^. Vaginal lavage sample were smeared onto a clear glass slide and stained with methylene blue. Vaginal cells were analysed by light microscopy in terms of morphological criteria. We assessed the length of each cycle as the length of time between two consecutive occurrences of oestrus and determined the time spent in each stage of the cycle.

### Serum preparation and hormone assays

Blood samples were gathered by enucleation while mice were in dioestrus. Then, samples were centrifuged at 3000 rpm for 15 min to collect the serum, which was stored at −80 °C. Serum oestradiol (E_2_) and follicle-stimulating hormone (FSH) levels were measured using a radioimmunoassay in the radiology department of the First Affiliated Hospital of Nanjing Medical University in accordance with the manufacturer’s instructions.

### H&E staining and follicle counting

Ovaries (*n* = 5 per group) were fixed with 4% paraformaldehyde overnight and then processed for histology according to standard protocols. Tissues were serially sectioned (4 µm thick) throughout the entire ovary, and every fifth section was placed on a clear glass slide and stained with haematoxylin and eosin (H&E). Follicle counting was performed as previously described with minor modifications^24^. In brief, to avoid counting a follicle twice, only follicles with a visible oocyte nucleus were counted. The following follicle classification system was used: primordial follicles were identified as those containing an oocyte surrounded by one layer of flattened granulosa cells; primary follicles were characterised by the presence of an enlarged oocyte surrounded by one layer of columnar granulosa cells; secondary follicles were defined as those containing more than one layer of columnar granulosa cells with no visible antrum surrounding the oocyte; antral follicles were identified as those with areas of follicular fluid or a single large antral space; and atretic follicles were identified as those exhibiting entry into a degenerative process without ovulation^25^. In atretic follicles, oocytes were shrunken or absent, and granulosa cells were replaced by fibrous material. The number of follicles in each category was counted by two experimenters without knowing the groups.

### Immunohistochemistry staining

Sections were obtained from each mouse ovary in the two groups of mice (*n* = 5 per group) and used for immunohistochemical analysis. First, paraffin sections were dewaxed, and heat-mediated antigen retrieval was then performed by microwaving the sections in 10 mM sodium citrate (pH 6.0) for 15 min. The sections were cooled to room temperature and rinsed three times with PBS. Experimental procedures were implemented according to the manufacturer’s instructions for the SP Rabbit & Mouse HRP Kit (DAB). The samples were incubated overnight with primary antibodies (Abs) against 8-hydroxyguanosine (8-OHdG), 4-hydroxynonenal (4-HNE), nitrotyrosine (NTY) and P16. Negative control samples were incubated with a biotin-conjugated goat antibody against rabbit IgG (H + L). The specimens were re-stained with haematoxylin for 30 s. All sections were incubated simultaneously with the same antibody concentrations under the same conditions. The sections were observed and imaged by microscopy, and staining was semi-quantified with ImageJ software. Staining was quantified by measuring the immunoreactive area (IA) in µm² and the integrated optical density (IOD). The staining intensity (SI) for each image was calculated as SI = IOD/IA.

### TUNEL staining

TUNEL of apoptotic cells in ovarian tissue was detected in paraffin sections using a One Step TUNEL Apoptosis Assay Kit following the manufacturer’s protocol. The sections were then counterstained with DAPI to visualise nuclei. TUNEL-positive cell counting was performed as previously described with minor modifications^26^. To eliminate histological differences between ovarian tissues, four random fields per slide (five slides per animal, five animals per group, *n* = 5) were examined. In total, 100 random fields (5 × 5 × 4 = 100) per group were examined. The TUNEL-positive granulosa cells and total granulosa cells in the antral follicles were counted. The rate of TUNEL-positive granulosa cells (%) in the antral follicles was analysed using ImageJ software.

### Western blotting

Western blotting was performed as described in a previous study^27^ In brief, protein quantification was performed using a BCA kit. Protein lysates obtained from ovarian tissues were subjected to 15% polyacrylamide gel electrophoresis and then electrotransferred to PVDF membranes. The membranes were blocked with a 5% BSA solution for 1 h and then incubated with the following primary antibodies in blocking buffer overnight at 4 °C: rabbit monoclonal anti-Prdx4, rabbit polyclonal anti-glucose-regulated protein 78 (GRP78), anti-activating transcription factor 4 (ATF4), anti-C/EBP homologous protein (CHOP), anti-activating transcription factor 6 (ATF6), anti-caspase-12, rabbit monoclonal anti-BAX, rabbit polyclonal anti-superoxide dismutase 1 (SOD1), anti-superoxide dismutase 2 (SOD2), anti-catalase (CAT) and anti-GAPDH. The membranes were washed three times with TBST before incubation with HRP-conjugated secondary antibodies. The membranes were then washed three times in TBST and imaged with an Alpha Imager after development with Pierce ECL Western Blotting Substrate. For quantification of the western blot analysis results, protein levels were normalised to GAPDH levels prior to normalisation to the controls. Quantitative results were analysed using ImageJ software.

### Statistical analysis

All graphs and statistical analyses were managed using GraphPad Software. Data in bar plots are shown as the mean ± SEM and were analysed using the Student’s *t* test or one-way ANOVA. A *P* value < 0.05 was considered statistically significant. And the investigator was blinded to the group allocation when assessing the outcome.

## Results

### Establishment of the Prdx4^−/−^ mouse model and determination of the appropriate d-gal concentration for ovarian ageing induction

**Fig. 1:**
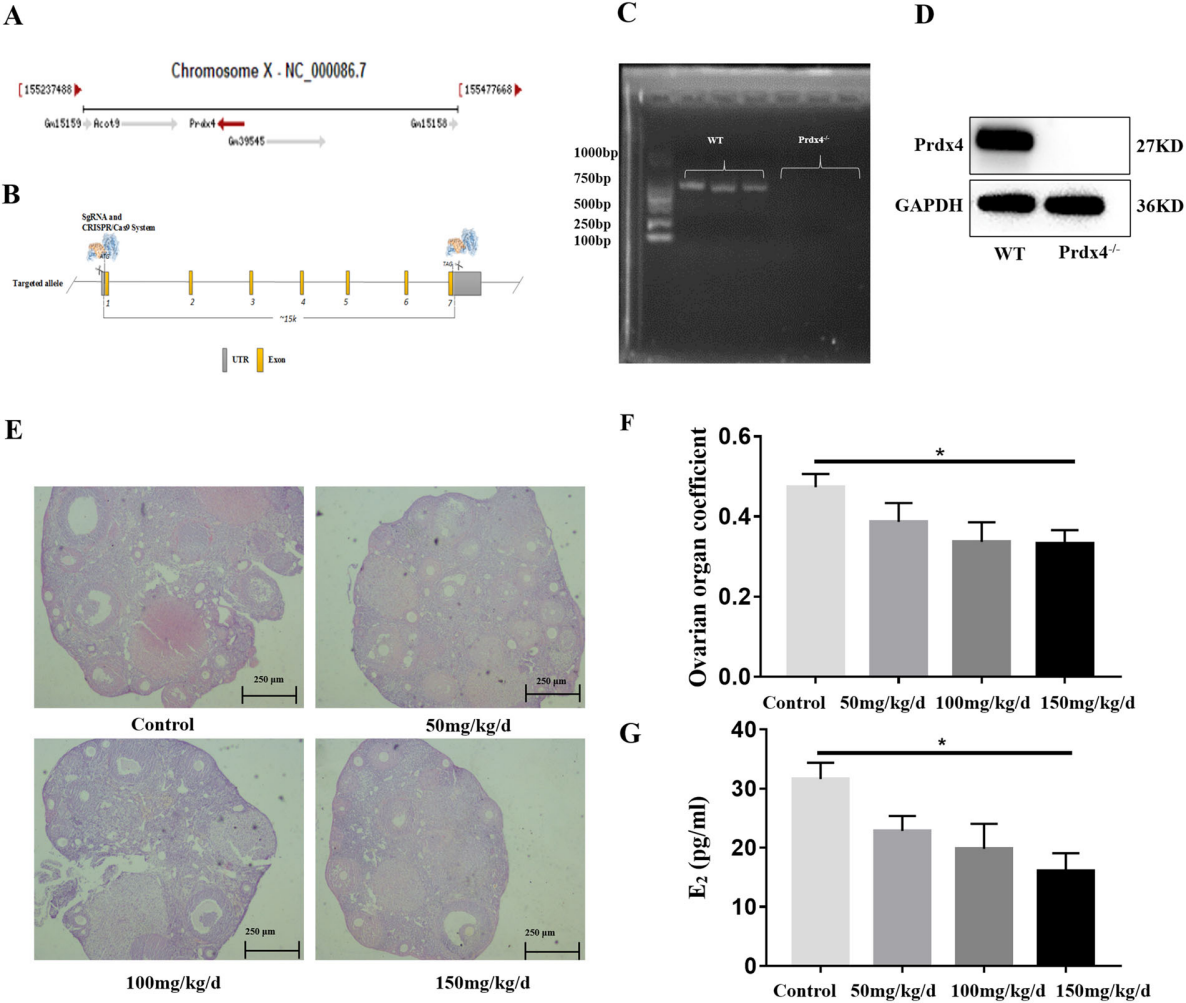
Establishment of the Prdx4^−/−^ mouse model and determination of the appropriate d-gal concentration for ovarian ageing induction. **a** The Prdx4 gene is located on the X chromosome. **b** Schematic illustration of Prdx4 exons and deletion of exons 1–7 by the CRISPR/Cas9 technique. **c** PCR genotyping of mice. PCR primers for the Prdx4 gene were as follows: forward, CCAGTATACAGTCTGATATGG, and reverse, TAGCTGGATATGTTAGCACT. These primers generated a 700-bp product. Genes from wild-type mice (lanes 1, 2, and 3) and Prdx4^−/−^ mice (lanes 4, 5, and 6) were amplified with specific primers. **d** Immunoblotting analysis of Prdx4 protein expression in the control and Prdx4^−/−^ groups. The blot was probed with an anti-Prdx4 antibody. GAPDH was used as the internal control. **e** The gross morphology of the ovaries of the four groups was observed by H&E staining. Scale bars = 250 μm at ×4 magnification. **f** Ovarian organ coefficient = ovarian weight/body weight (mg/g). **g** Serum E_2_ levels of the four groups were measured by radioimmunoassay. All data are shown as means ± SEMs, *n* = 5. Statistical significance: *P < 0.05.

The *Prdx4* gene is located on the X chromosome (Fig. 1a). Transcription of the *Prdx4* gene generates five different transcripts, the longest of which is 100 bp in length and contains 7 exons. To generate Prdx4^−/−^ mice by CRISPR/Cas9-mediated genome editing, the gRNA directs Cas9 endonuclease-mediated cleavage of the *Prdx4* gene and creates a double-strand break (DSB). Such breaks are repaired, resulting in deletion of E1-E7 (Fig. 1b). Mouse tail DNA was extracted for PCR, and ovarian tissue protein was extracted for western blotting to identify the genotypes of mice (Fig. 1c, d).

The numbers of follicles in each of the various follicle categories, the ovarian organ coefficient and the serum E_2_ level in the high-dose group (d-gal, 150 mg/kg/day) were significantly reduced compared with those in the control group (*P* < 0.05, Fig. 1e–g). Therefore, we selected 150 mg/kg/day of d-gal as an appropriate concentration to establish the mouse model of ovarian ageing in this study.

### Acceleration of reproductive ageing in Prdx4^−/−^ mice

**Fig. 2:**
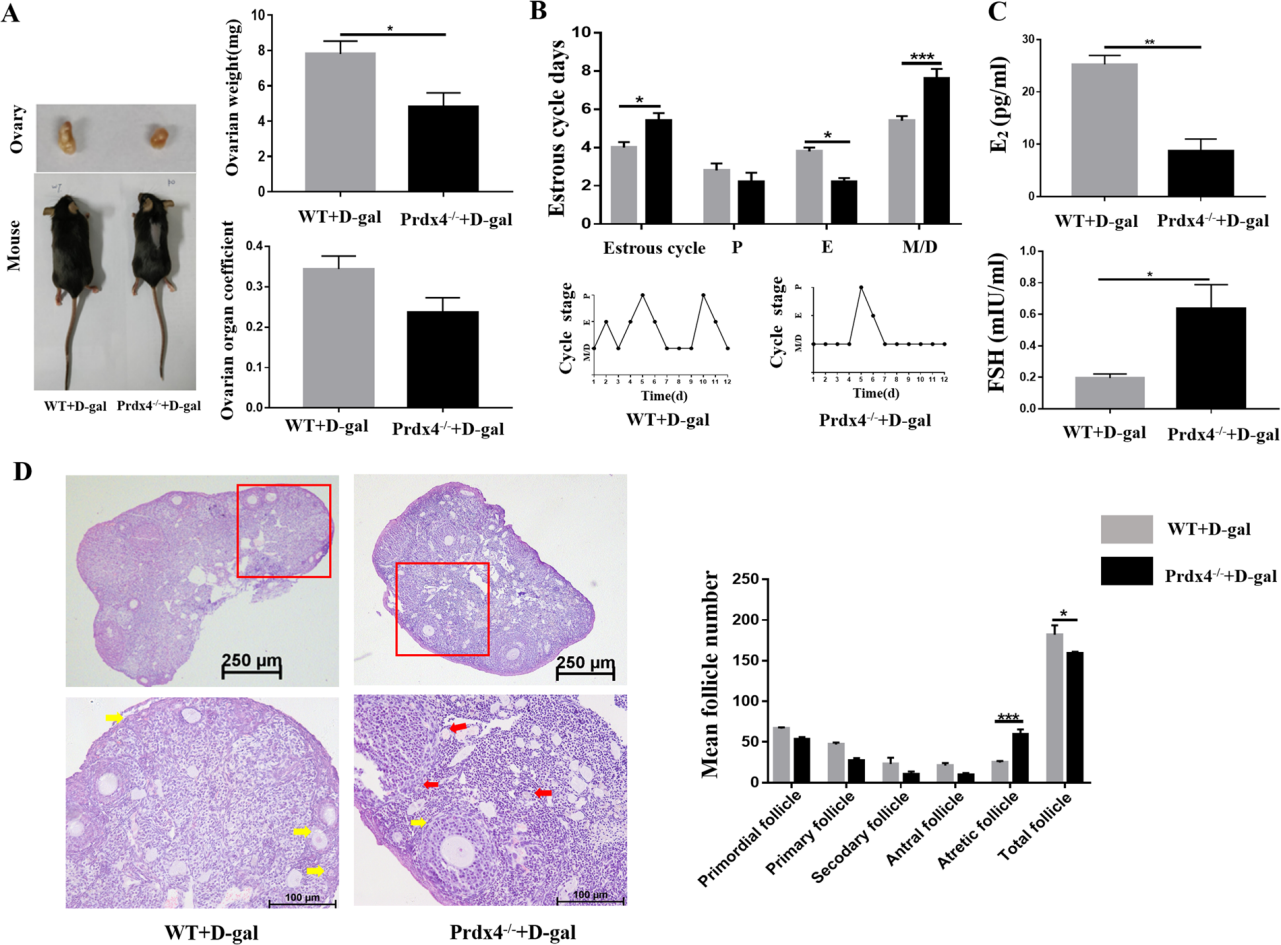
Acceleration of reproductive ageing in Prdx4^−/−^ mice. WT and Prdx4^−/−^ mice were injected with d-gal (150 mg/kg/d) for 42 consecutive days (*n* = 5 mice per group). **a** Representative images of ovaries and mice from the two groups are shown. There was a significant difference in ovarian weight between the two groups and a substantial decrease in ovarian organ coefficient in the Prdx4^−/−^ group. **b** The mean durations of the oestrous cycle and number of days spent in pro-oestrus (P), oestrus (E), metoestrus (M), and dioestrus (D) were evaluated by vaginal cytology over the course of 12 consecutive days. The representative oestrous cyclicity in each treatment group is presented. **c** Serum E_2_ and FSH levels during dioestrus were measured in the two groups. **d** Histology of ovarian sections from the two groups. The yellow arrows show the primordial, primary, or secondary follicles, and the red arrows indicate the developmentally arrested follicles with degenerating oocytes. Scale bars = 250 μm at ×4 magnification; scale bar = 100 μm at ×20 magnification. The number of follicles at different developmental stages is summarised. All data are shown as means ± SEMs, *n* = 5. Statistical significance: *P < 0.05, **P < 0.01, ***P < 0.001.

Analysis of the gross morphology of the two groups of mice showed no significant differences, but weight loss occurred slightly faster in the Prdx4^−/−^ group than in the WT group when the POF model was induced by d-gal. Compared with the ovarian weight in the WT group, the ovarian weight in the Prdx4^−/−^ group was significantly decreased (*P* < 0.05), but the ovarian organ coefficient was not significantly decreased (Fig. 2a). The hypothalamic-pituitary-ovary (HPO) axis was more strongly disrupted in the Prdx4^−/−^ group than in the WT group. Oestrous cyclicity was examined via a daily vaginal smear. The results showed that the control group exhibited regular oestrous cycles with a duration of 4–5 days; however, the Prdx4^−/−^ group showed irregular oestrous cycles with an obviously decreased length of the oestrous period and an increased length of the dioestrus period (Fig. 2b). We then collected mouse sera to analyse the levels of E_2_ and FSH, and the Prdx4^−/−^ group showed a significant decrease in the serum E_2_ level (*P* < 0.01, Fig. 2c) and an increase in the serum FSH level (*P* < 0.05, Fig. 2c). Follicle counting was performed after H&E staining. In Prdx4^−/−^ mice, the numbers of primordial, primary, secondary and antral follicles were relatively reduced, but the number of atretic follicles was obviously increased (*P* < 0.001, Fig. 2d).

### Increased ovarian cell apoptosis in Prdx4^−/−^ mice

**Fig. 3:**
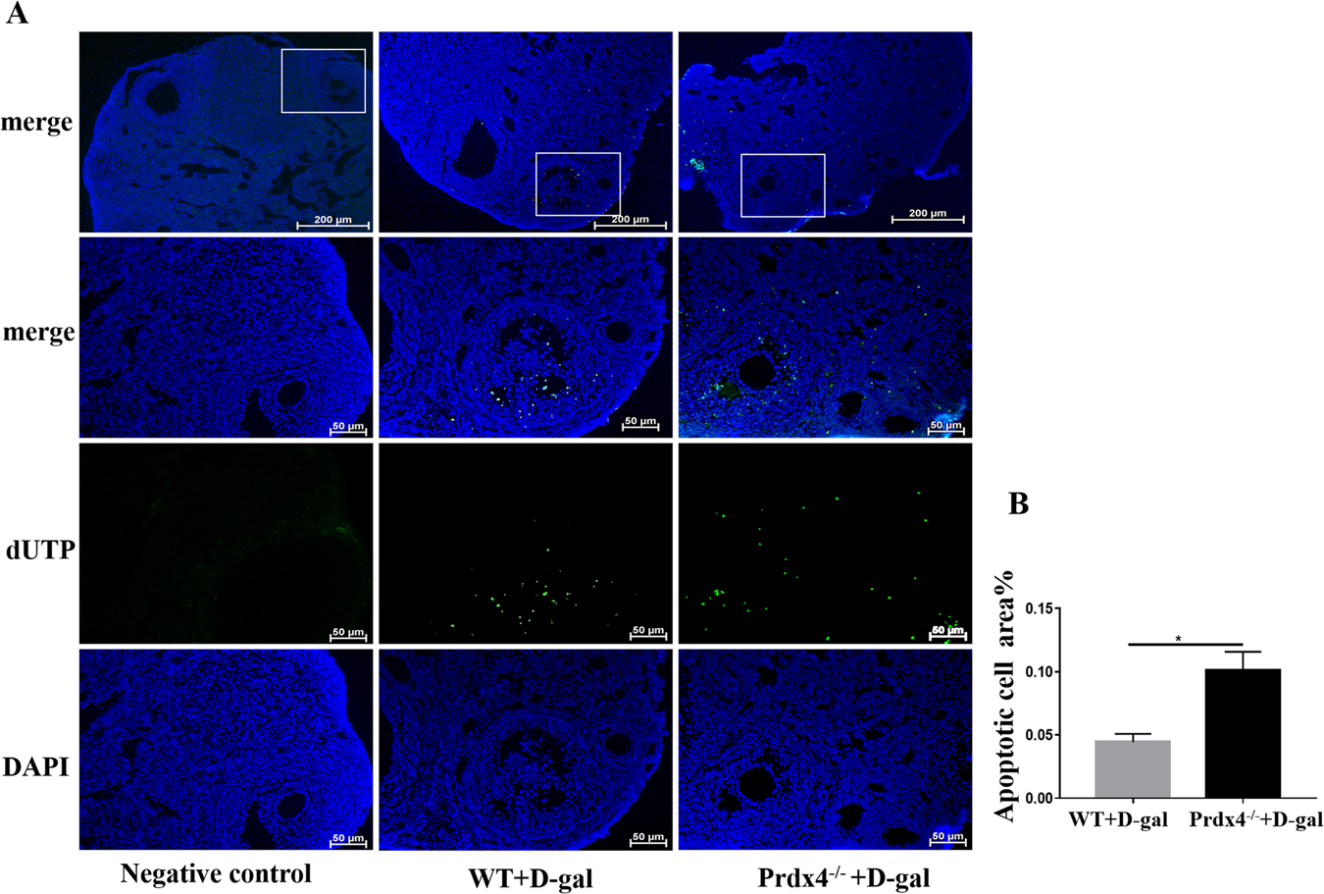
Increased ovarian cell apoptosis in Prdx4^−/−^ mice. **a** Apoptosis was analysed using an in situ TUNEL fluorescence assay (×20). In the TUNEL assay, nuclei of TUNEL-positive (apoptotic) cells were stained green. **b** The number of TUNEL-positive granulosa cells was compared between the two groups. Scale bars = 200 μm at ×10 magnification; scale bar = 50 μm at ×20 magnification. All data are shown as means ± SEMs, *n* = 5. Statistical significance: *P < 0.05.

We observed visible green-stained nuclei of TUNEL-positive (apoptotic) granulosa cells in antral follicles but not in the primordial and primary follicles (Fig. 3a). The number of apoptotic granulosa cell areas was assessed in the two groups with ImageJ software. More apoptotic cells were detected in the Prdx4^−/−^ group than in the control group (*P* < 0.05, Fig. 3b).

### Increased oxidative damage and ovarian senescence-associated protein expression in Prdx4^−/−^ mice

**Fig. 4:**
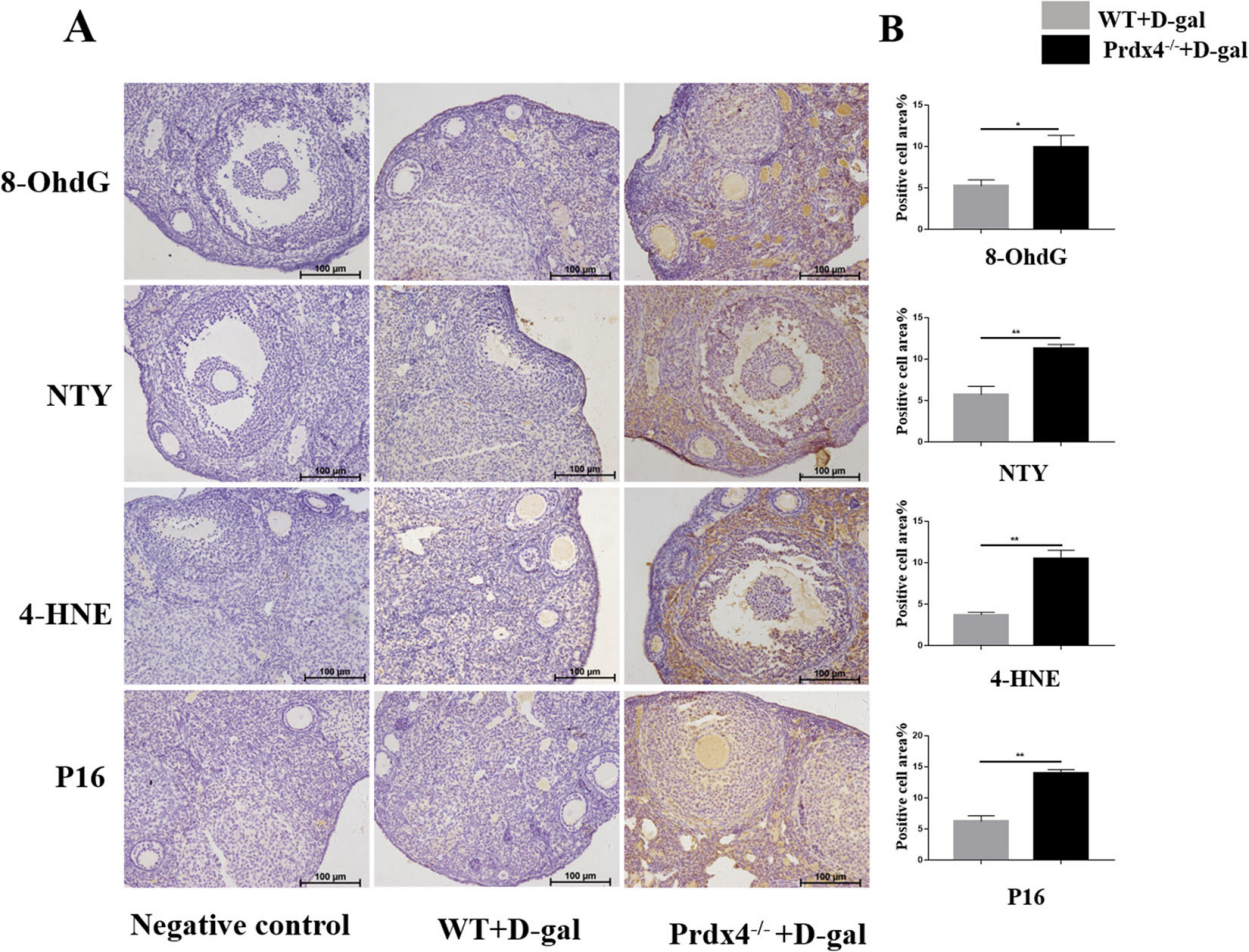
Increased oxidative damage and ovarian senescence-associated protein expression in Prdx4^−/−^ mice. **a** The cellular localisation of oxidative damage-related factors (8-OHdG, NTY and 4-HNE) and an ovarian senescence-associated protein (P16) were observed using immunohistochemistry. The Prdx4^−/−^ group showed significantly increased expression of 8-OHdG, NTY and 4-HNE proteins in ovarian interstitial cells. The ovarian senescence-associated P16 protein was localised mainly in granulosa cells and oocytes, and their expression levels were relatively decreased in interstitial cells. Obviously increased P16 expression was detected in the Prdx4^−/−^ mouse group. Scale bars = 100 μm at ×20 magnification. **b** Protein expression was quantitatively analysed. All data are shown as means ± SEMs, *n* = 5. Statistical significance: *P < 0.05, **P < 0.01.

Oxidative DNA, protein, and lipid damage and the level of the senescence-associated protein P16 in interstitial cells and follicles were examined using immunohistochemistry. The 8-OHdG, NTY and 4-HNE proteins were localised mainly in ovarian interstitial cells. Compared with the control group, the Prdx4^−/−^ group showed significant increases in 8-OHdG, NTY and 4-HNE protein immunostaining (*P* < 0.05, Fig. 4a, b). The ovarian senescence-associated P16 protein was localised mainly in granulosa cells and oocytes, and its expression levels were relatively decreased in interstitial cells. Significantly increased P16 expression was detected in the Prdx4^−/−^ mouse group (*P* < 0.01, Fig. 4a, b).

### The expression of compensatory antioxidant enzymes and ER stress pathway-associated factors in the ovary

**Fig. 5:**
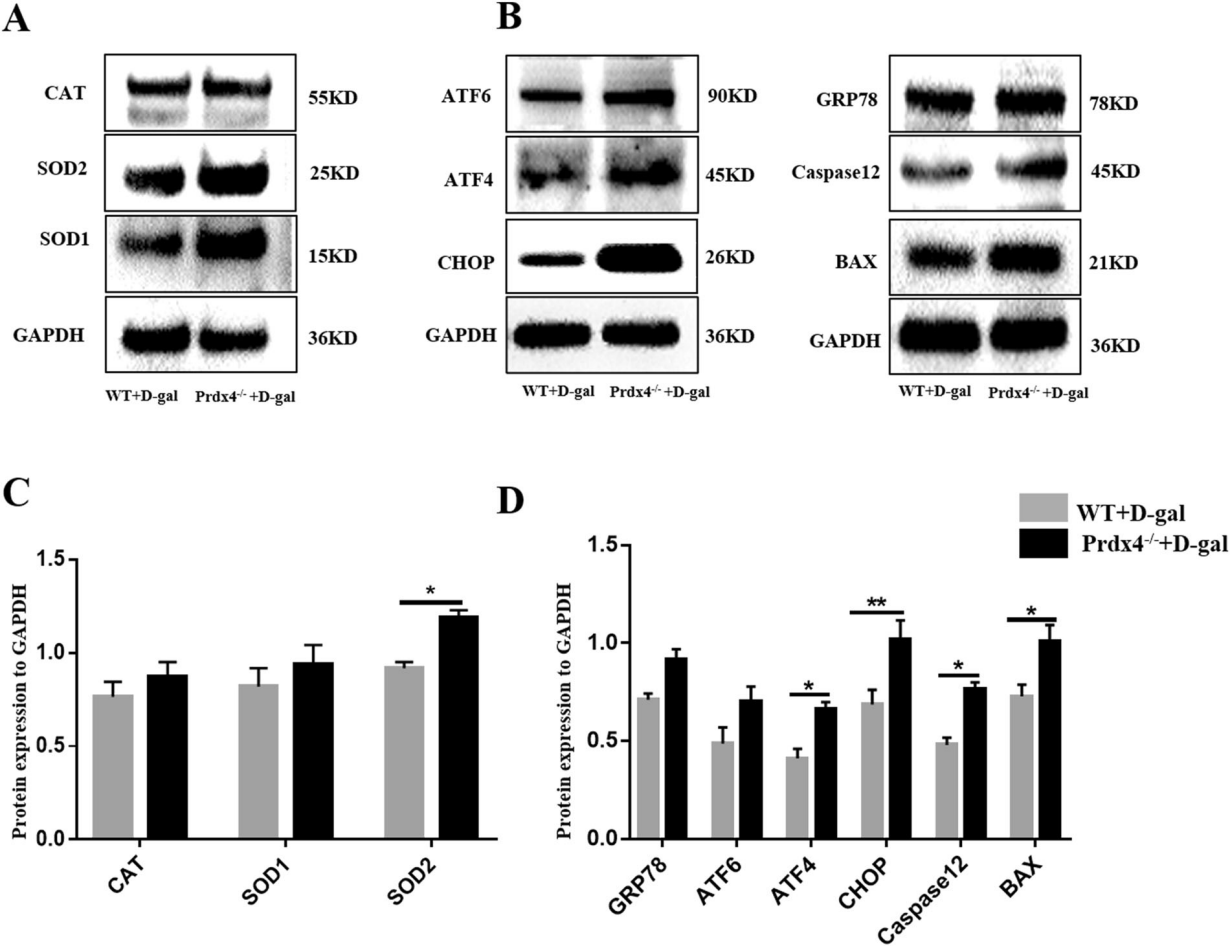
The expression of compensatory antioxidant enzymes and ER stress pathway-associated factors in the ovary. **a** The CAT, SOD1 and SOD2 protein expression levels were measured by western blotting. **b** The levels of ER stress pathway-related factors (GRP78, ATF6, ATF4, CHOP, and caspase-12) and induced expression of the apoptotic protein BAX in ovarian tissues were evaluated using western blotting. **c** The CAT, SOD1, and SOD2 protein expression levels were quantitatively analysed. **d** The GRP78, ATF6, ATF4, CHOP, caspase-12, and BAX protein expression levels were quantitatively analysed. All data are shown as means ± SEMs, *n* = 5. Statistical significance: *P < 0.05, **P < 0.01.

In Prdx4^−/−^ mice, the levels of oxidative damage-related factors were visibly increased, as assessed by immunohistochemistry. In addition, the expression of other compensatory antioxidant enzymes (CAT, SOD1 and SOD2) was examined by western blotting (Fig. 5a). CAT and SOD1 protein expression levels were relatively higher in Prdx4^−/−^ mice than in WT mice, and the SOD2 protein expression level was significantly higher in Prdx4^−/−^ mice (*P* < 0.05, Fig. 5c). Prdx4 was localised to the ER as determined by colocalization with calreticulin^16^. The expression levels of ER stress signalling pathway-associated markers were assessed by western blotting (Fig. 5b, d). The expression level of an ER stress marker (GRP78) was relatively increased in the Prdx4^−/−^ group. We examined the regulation of 2 major components of the unfolded protein response (UPR) pathways, namely, ATF6 and ATF4, and found that the ATF4 expression level was significantly increased (*P* < 0.05). The expression levels of CHOP/GADD153 and caspase-12 (regulators and markers of ER stress-induced apoptosis) were significantly increased (*P* < 0.05). Upregulation of the proapoptotic gene BAX (*P* < 0.05) induced apoptosis in the Prdx4^−/−^ mouse group.

## Discussion

Many studies have shown that d-gal directly induces oxidative stress in vivo and that galactose toxicity inhibits E_2_ production from granulosa cells^28^. In this study, we found that the level of serum E_2_ and the numbers of primordial, primary and secondary follicles were significantly decreased in mice treated with d-gal (Fig. 1e, g). Thus, the use of d-gal successfully induced the mouse POF model in the present study. The d-gal-treated mouse ageing model in this research not only partially simulated ageing-related physiological senescence in the body, but also facilitates an understanding of the pathogenesis of ovarian ageing caused by a large amount of oxidative stress. Many factors, including iatrogenic factors, harmful environments and some pathological events, can cause a sharp decline in ovarian function due to oxidative damage. Therapeutic processes such as radiotherapy and chemotherapy in cancer patients and ischaemia and reperfusion during ovarian surgery impair the ovaries through oxidative stress-induced apoptosis of follicular cells^29^. Exposure to many toxic chemicals, such as those in cigarettes and pesticides, polycyclic aromatic hydrocarbons and cypermethrin, in residential and occupational environments reduces ovarian function by generating additional ROS in vivo^30^. Obesity and abnormal lipid metabolism are associated with infertility and high oxidative levels in the ovarian microenvironment, which may play a role in the pathogenesis of disease-related infertility^31^. These forms of acute and severe oxidative damage may cause a rapid decline in ovarian function and lead to a POF. Therefore, accelerating ovarian ageing with d-gal is of practical significance.

Prdx4 is an antioxidant, anti-steatosis, anti-inflammatory and anti-apoptotic substance^32^. As revealed by our previous work, Prdx4 expression closely follows follicular development^19^. This study was designed to investigate the protective effect of Prdx4 on ovarian ageing induced by d-gal. Ovarian ageing is a major factor in female infertility and characterised by decreased follicular quantity and quality^33^. Our data showed that with d-gal treatment, Prdx4 deficiency accelerated ovarian ageing, including increased follicular atresia and HPO axis disorders. Many studies strongly support the idea that antral follicular atresia is caused mainly by granulosa cell apoptosis^34^. Granulosa cells are essential for follicle development and homeostasis because they provide nutrients and mechanical support for oocytes via physical interactions. As revealed in our previous work, Prdx4 expression was mainly located in granulosa cells. We also detected increased apoptosis of ovarian granulosa cells in Prdx4^−/−^ mice (Fig. 3), leading to an increase in follicle attrition and a decrease in steroid hormone production^35^.

To our knowledge, mammalian cells contain six peroxiredoxins, but only Prdx4 is localised to the ER. Prdx4 exerts two advantageous effects in the ER: promotion of protein disulphide bond synthesis and suppression of oxidative stress by eliminating H_2_O_2_^36^. Prdx4 shows very strong reactivity with H_2_O_2_ in the ER lumen^37^, and excessive production of H_2_O_2_ is thought to lead to oxidative stress. Excess ROS lead to a wide range of oxidative damage in cell structures, including lipid peroxidation and DNA damage as well as membrane and protein oxidation, ultimately driving cell death^38^. Additionally, Prdx4 deficiency leads to increases in misfolded proteins and H_2_O_2_ in the ER lumen, inducing ER stress^39^. Furthermore, studies have demonstrated that when using a dextran sulphate sodium (DSS)-induced colitis model, lack of Prdx4 aggravates the colonic mucosal damage caused by ER stress^40^. Prdx4 functions as an ER thiol oxidase and antioxidant and protects the homeostasis of the cell environment. In our study, the alterations of oxidative damage- and ER-stress-related biomarkers confirmed that when all mature mice were treated with d-gal, Prdx4 deficiency increased unfavourable ROS-mediated sulfhydryl oxidation in proteins and triggered ER stress. The evidence strongly indicates that ROS resulting from exposure to specific chemical or physical agents are involved in the initiation of granulosa cell apoptosis^41^. Many studies have demonstrated that severe and persistent ER stress plays a crucial role in regulating GC cell apoptosis^42^. Thus, we concluded that Prdx4 may protect against ovarian ageing by reducing ovarian granulosa cell apoptosis via inhibiting oxidative stress and ER stress-related pathways. Although the specific mechanism has not been fully elaborated, these results provide further evidence supporting the significant role of Prdx4 in protecting ovarian function from ROS damage and ER stress.

Interestingly, we found that P16 expression in follicles was significantly increased in Prdx4^−/−^ mice (Fig. 4). P16 protein negatively regulates cell proliferation and division and promotes apoptosis and senescence. And, it affects the cell cycle and G1-S conversion by competitively suppressing the interaction between CDK4/6 and cyclin D within G1 phase^43^. The P16 expression level was positively related to the follicular number and negatively related to E_2_ and P levels^28^. P16 expression was low in young animal tissues and subsequently increased with age^44^. Baker et al.^45^ found that aging cells were deactivated and ageing-associated phenotypes reduced by silencing P16 protein expression. However, how P16 participates in ovarian aging through oxidative stress is unclear. We observed an important effect of P16 on oxidative stress in ovarian follicles and speculated that Prdx4 deficiency also accelerated ovarian ageing partially through the upregulation of P16.

In summary, our current findings suggest that Prdx4 deficiency accelerates ovarian ageing, including increased follicle atresia and HPO axis disorders, in mice exposed to d-gal-induced oxidative damage. Prdx4 may protect against ovarian ageing by suppressing ovarian granulosa cell apoptosis via multiple mechanisms, including inhibiting oxidative stress and ER stress-related pathways. These results provide an understanding of the pathogenesis of ovarian ageing caused by a large amount of oxidative stress. Based on the downregulated Prdx4 expression observed in premenopausal ovaries in our previous clinical study, we will further clarify the role of Prdx4 in sustaining female reproduction to reveal new diagnostic biomarkers and potential therapeutic targets for use in patients with POF.

## Supplementary information

KEY RESOURCES TABLE
