# Supplementary material for: Peroxiredoxin 4 protects against ovarian ageing by ameliorating d-galactose-induced oxidative damage in mice
Source: Cell Death Dis. 2020 Dec 11;11(12):1053. doi: 10.1038/s41419-020-03253-8 (PMC7732846; doi:10.1038/s41419-020-03253-8)
Supplement: Supplementary file 1 — KEY RESOURCES TABLE [file 41419_2020_3253_MOESM1_ESM.docx]

| Reagent | Source | Identifier | |
| --- | --- | --- | --- |
| D-galactose | Sigama, America | G0750 |  |
| Methylene blue | Shanghai Yuanye Bio-Technology | S19043 |  |
| FSH | Beijing North Institute of Biotechnology | KIP0841 |  |
| E_2_ | Beijing North Institute of Biotechnology | KIP0629 |  |
| SP Rabbit & Mouse HRP Kit  TUNEL Apoptosis Assay Kit  ECL Wb Substrate | Kangwei Century Biotechnology  Beyotime Institute of Biotechnology  Thermo Scientific, USA | CW2069S  C1088  32106 |  |

**KEY RESOURCES TABLE**

| Antibodies | Source | Identifier | Concentration | |
| --- | --- | --- | --- | --- |
| Anti-8-OHdG | Abcam, USA | ab62623 | 1:200 | |
| Anti-4-HNE | Abcam, USA | ab46545 | 1:100 | |
| Anti-NTY | Abcam, USA | ab7048 | 1:200 | |
| Anti-P16 | Abcam, USA | ab189034 | 1:200 | |
| goat Ab against rabbit IgG  anti-Peroxiredoxin4  anti-GRP78  anti-ATF4  anti-CHOP  anti-ATF6  anti-caspase-12  anti-BAX  anti-SOD1  anti-SOD2  anti-CAT  anti-GAPDH | Beyotime, China  Abcam, USA  Cell Signaling Technology  Abcam, USA  Cell Signaling Technology  Santa Cruz  Abcam, USA  Abcam, USA  Abcam, USA  Proteintech  Santa Cruz Biotechnology  Santa Cruz Biotechnology | A0277  ab184167  3183  ab-23760  2895  sc-166659  ab62484  ab32503  ab13498  241271-AP  sc-13584  sc-32233 | | 1:200  1:1000  1:500  1:1000  1:1000  1:500  1:1000  1:2000  1:2000  1:1000  1:2000  1:2000 |
